# Pitfalls in the radiological response assessment of immunotherapy

**DOI:** 10.1007/s12254-018-0389-x

**Published:** 2018-03-21

**Authors:** Lucian Beer, Maximilian Hochmair, Helmut Prosch

**Affiliations:** 1 Department of Biomedical Imaging and Image-guided Therapy, Medical University of Vienna, Währinger Gürtel 18–20, 1090 Vienna, Austria; 2 Respiratory Oncology Unit, Otto-Wagner Hospital, Vienna, Austria

**Keywords:** Immune checkpoint inhibitor, PD-1 inhibitor, Pseudoprogression, Hyperprogression, Pneumonitis

## Abstract

Immunotherapies comprise of a class of cancer therapies that are increasingly used for treatment of several cancer entities. Active immunotherapies encompassing immune checkpoint inhibitors are the most widespread class of immunotherapies, with indications for melanoma, non-small lung cancer, renal cell carcinoma, urothelial carcinoma, head and neck squamous cell carcinoma, and Hodgkin’s lymphoma. Immune checkpoint inhibitors have demonstrated unique response patterns that are not adequately captured by traditional response criteria such das the Response Evaluation Criteria in Solid Tumors (RECIST) and World Health Organization criteria. Consequently, adaptions of these criteria have been released such as the immune-related RECIST and immune RECIST, which account for the specialities of immunotherapies. Immunotherapies can cause a distinct set of adverse events such as pneumonitis, colitis, and hypophysitis. In addition, atypical treatment response patterns termed pseudoprogression have been observed. Thereby, new or enlarging lesions appear after treatment start and mimic tumor progression, which is followed by an eventual decrease in total tumor burden. In this review article we will describe pitfalls in the radiological response assessment of immunotherapies, focusing on pseudoprogression and imaging appearances of common immune-related adverse events.

## Cancer immunotherapy

For decades oncologists have used cytotoxic chemotherapeutics that directly kill tumor cells for anticancer treatment. Growing knowledge of cancer development and its underlying immunological mechanisms has led to the development of immunotherapies. To prevent the development of malignancies, the immune system is able to identify tumor-associated antigens and remove the identified neoplastic cells [1]. A loss of immunological reactivity to neoplastic cells is a hallmark step in the development of cancer that leads to the continued growth and ability to spread in the body. Immunotherapies take advantage of the body’s own antitumor activity and boost its activity to mount a more effective antitumor response.

Based on their mode of action, immunotherapies can be described as active or passive in nature. Whereas active immunotherapies activate humoral or cellular mediated immunity, passive immune therapies exert antitumor activity via the clearance of tumor cells by binding to passively applicated preformed antibodies or other immune system components.

Especially active immunotherapies have been recently in focus, as many new immunomodulatory drugs of this class have been approved for treatment of several different tumor entities in the past years. In 2011 the immune checkpoint inhibitor ipilimumab—approved for treatment of metastatic melanoma—marked the start of a revolution in anticancer treatments that was followed by the approval of immune-checkpoint inhibitors for the treatment of non-small cell lung cancer (NSCLC), renal cell cancer, urothelial carcinoma, head and neck cancer, and Hodgkin’s lymphoma in various stages [2–5]. Multiple other malignancies (e. g., gastric cancer, hepatocellular cancer, ovarian cancer, mesothelioma) are currently under clinical investigation to evaluate the potential benefit of these drugs [6]. Immunotherapies are not only being used in clinical trials and as second- or third-line therapies, but also as a first-line treatment option [7]. This highlights the necessity not only for radiologist, but also for clinicians to become familiar with the characteristics of radiological response assessment.

## Radiological response assessment

The most commonly used response assessment criteria for classical chemotherapeutics are the World Health Organization (WHO) criteria and Response Evaluation Criteria in Solid Tumors (RECIST) 1.0 published in 2000 and its update RECIST 1.1, released in 2009 [8]. Both classifications take into account morphological changes during therapy, whereas an increase in tumor size and/or the appearance of new lesions are seen as progressive disease (PD) and indicate treatment failure.

However, response patterns using immunotherapies can differ significantly to those from classical chemotherapies and an increase in tumor size and/or appearance does not always represent disease progression, but also can be a result of antitumor activity-driven immune cell infiltration and thus treatment response. Based on clinical data of 487 patients with advanced melanoma treated with ipililumab, a novel response pattern has been described and incorporated into the so-called immune related response criteria (irRC) [9]. Basically, four different forms of treatment response have been reported.

1. Reduction in tumor size after treatment initiation in comparison to baseline.
2. Initial increase of tumor size and/or new lesions followed to a decrease that meets criteria for partial or complete response in comparison to baseline.
3. Initial increase in tumor size and/or new lesions followed by a stable course.
4. Almost stable tumor size without any significant changes.

Whereas scenario one isn’t challenging for radiologists and clinicians, scenarios two and three can be easily misinterpreted as treatment failure using classical response criteria. These latter two phenomena are often referred to as “pseudoprogression” and are characterized by an initial increase of tumor burden and/or appearance of new lesions followed by subsequent decrease or stabilization of tumor burden [1, 10, 11].

## Pseudoprogression

Pseudoprogression is a relatively uncommon phenomenon with an incidence of 4 to 10% in melanoma patients [5, 9, 10] and only 0.6 to 5% in NSCLC patients [12, 13] treated with immune checkpoint inhibitors. Therefore, in most cases, an increase of tumor size is due to treatment failure and true progression rather than being a pseudoprogression. In melanoma patients it has been shown that this phenomenon can occur in lymph nodes, but more commonly in non-nodal locations such as the kidneys, liver, lungs, peritoneum, adrenal gland, and chest and abdominal wall [14].

Pseudoprogression is challenging for both radiologists and clinicians, and, to date, there is no valid biochemical or radiological marker that can help to differentiate between true progression or hyperprogression and pseudoprogression [15].

Radiologically, although more frequently seen in the first weeks after treatment initiation, pseudoprogression can also be seen months after treatment initiation [16]. In addition, as pseudoprogression can lead to an increased metabolic activity, positron emission tomography (PET) imaging hampers reliable identification of pseudoprogressors.

The irRC as well as a modification of RECIST 1.1 for immune-based therapeutics (iRECIST) that were published in 2017 [17] were primarily designed for standardization of reporting and data collection in clinical trials, but not for routine clinical use and therefore helpful in an only limited field. Exemplarily, both criteria recommend a follow-up imaging to prove or rule out progression at 4 to 8 weeks after first progression. In Table 1 the characteristics of the RECIST 1.1, irRC, and iRECIST criteria are given (adapted to [9, 17]).

**Table 1:** Comparison of RECIST 1.1, irRC and iRECIST

	RECIST 1.1	irRC	iRECIST
Complete Response	Disappearance of all target lesions or lymph nodes <10 mm in the short axis	Disappearance of all target lesions or lymph nodes in 2 consecutive observations not less than 4 weeks apart	Disappearance of all target lesions or lymph nodes <10 mm in the short axis
Partial Response	>30% decrease in tumor size or ≥15% decrease in tumor attenuation at CT, no new lesions	≥50% decrease in tumor burden compared with baseline in 2 observations at least 4 weeks apart	>30% decrease in tumor size or ≥15% decrease in tumor attenuation at CT, no new lesions
Progressive Disease	>20% increase of SPD of target lesions with an absolute increase of ≥5 mm, new lesions	≥25% increase of SLD compared with nadir (at any singe time point) in 2 consecutive observations at least 4 weeks apart	Differentiation between iUPD and iCPD (see below), iUPD can result in PR or CR
Stable Disease	None of the above	None of the above	None of the above
New Lesions	Results in PD	Results in PD that has to be confirmed in 2 observations at least 4 weeks apart	Results in iUPD and consequently in iCPD if additional new lesions appear or an increase of size of new lesions (>5 mm for SLD or any increase of non-target lesions)
Confirmation of PD	Not required (unless equivocal)	Required	Required
Consideration of clinical status	Not included in assessment	Not included in assessment	Clinical stability is considered in whether treatment is continued after iUPD

*RECIST 1.1.* Response Evaluation Criteria in Solid Tumors; *irRC* Immune-Related RECIST; *iRECIST* immune RECIST; *PD* progressive disease; *CR* complete response; *SD* stable disease; *iUPD* unconfirmed immune PD; *iCPD* confirmed immune PD; *SPD* sum of the products of diameters

**Fig. 1:**
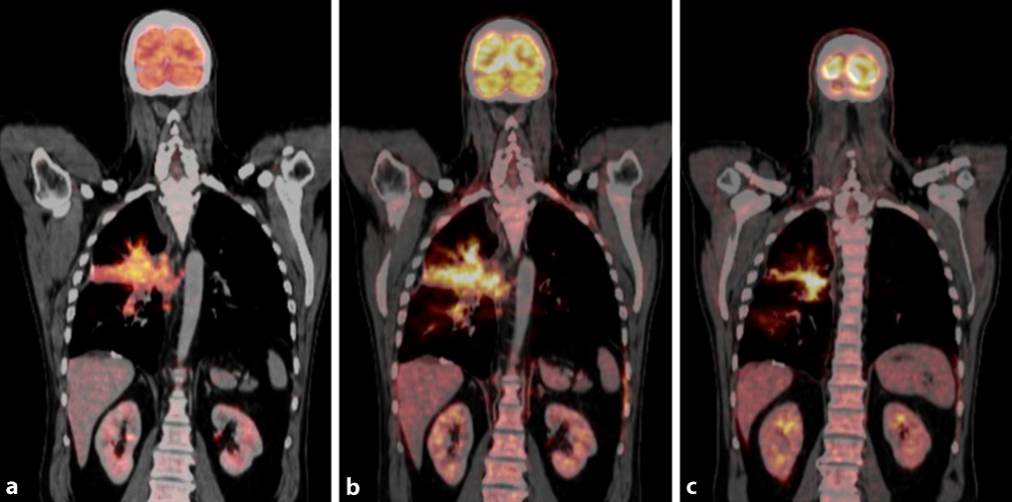
Pseudoprogression in 54-year-old man with non-small cell lung cancer receiving immune checkpoint inhibitor therapy. **a** Coronary fluorodeoxyglucose positron emission tomopraphy/computed tomography (^18^F-FDG-PET/CT) imaging obtained before therapy demonstrate ^18^F-FDG avid malignant tumor in the right lung. **b** 5 weeks after treatment initiation tumor size and FDG uptake increased. Therapy was continued and 6 weeks thereafter tumor size shrinkage and a reduced ^18^F-FDG uptake were observed (**c**)

However, clinical data show that subsequent progression in size can even last for 12 weeks before starting to decrease [5]. Therefore, a follow-up scan at 4 weeks or even 8 weeks might be too early to rule out pseudoprogression and longer follow-up intervals are needed (Fig. 1). Further studies with larger numbers of patients that address these limitations of the current immune-related response criteria will hopefully yield guidelines for routine clinical use.

An initial increase of tumor burden followed by stabilization can be seen as a form of pseudoprogression and clinically described as “treatment beyond progression.” This can be the case in patients treated with tyrosine kinase inhibitors (TKI) who benefit from continuing treatment even in radiological PD [18], as well as in patients with immune checkpoint inhibitors who do not show worsening of their performance status.

## Hyperprogression

Contrary to the above-mentioned conditions in which treatment continuation—although radiological disease progress—is beneficial, patients with immunotherapies can experience a severe progression of tumor burden referred as to hyperprogression. Hyperprogression is defined as an increase in tumor growth rate after treatment initiation by a factor of two [19, 20]. According to Champiat et al., up to 9% of patients with different histological tumor types treated with immune checkpoint inhibitors develop hyperprogression, which is associated with worse overall survival [20]. In general, hyperprogression more commonly effects elderly patients (<65 years old), without any a difference between histology’s of cancers including melanoma, colorectal, ovarian, biliary tract, urothelial carcinomas, and lymphomas [20].

A similar response pattern has been described after discontinuation of TKI therapies in patients with EGFR-mutant lung cancer and was termed “disease flare”[21, 22].

## Imaging of immune-related adverse events

Radiologists must be able to recognize the unique spectrum of immune-related adverse reactions (irAE). In comparison with cytotoxic chemotherapy, irAEs are attributed to autoimmunity and infiltration of autoreactive T cells. They can occur in almost all organs and may be first identified on CT or fluorodeoxyglucose F18 (^18^F-FDG)-PET/CT performed for restaging and/or surveillance imaging. ^18^F-FDG-PET/CT is deemed to be more sensitive for the detection of irAEs and is able to identify them earlier than CT.

The most common irAEs are dermatologic, including vitiligo, rash, and erythema. irAEs detectable in radiological examinations are colitis, pneumonitis, thyroiditis, hepatitis, pancreatitis, hypophysitis, and arthritis [23, 24].

Colitis is more common in patients treated with ipililumab (8–38%) than in patients treated with nivolumab or pembrolizumab (1–20%) [25], and occurs typically 6–7 weeks after initiation of treatment. Three distinct types of immune-mediated colitis have been described, encompassing a diffuse colitis, segmental colitis, and an isolated rectosigmoidal colitis. Image findings of such are non-specific and include mesenteric hyperemia, bowel wall thickening, increased mucosal enhancement, and fluid-filled small or large bowel [26]. Imaging findings of hepatitis are also non-specific and similar to those seen in acute hepatitis including hepatomegaly, periportal tracking, reduced density in comparison to baseline, and periportal lymphadenopathy. Immune-mediated pancreatitis is rare, with an incidence below 1%. Imaging findings are an organomegaly, reduced density of the pancreas, and increased density of the peripancreatic fat. In ^18^F-FDG-PET/CT an increased FDG uptake had been described [23].

**Fig. 2:**
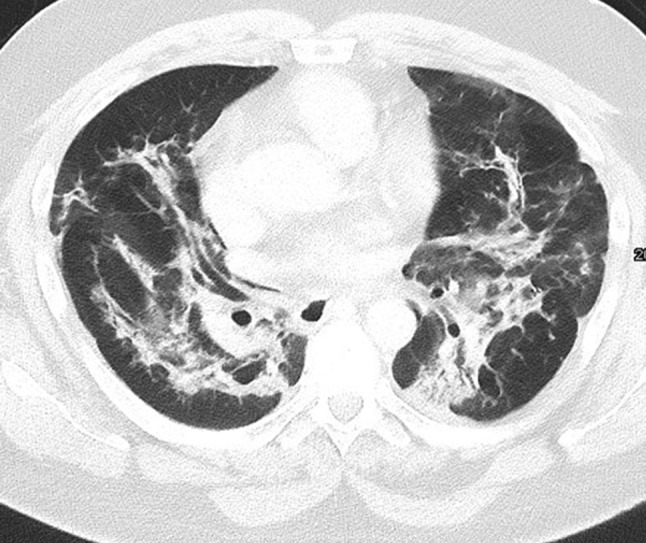
Organizing pneumonia in a 53-year-old man with epithelial cell carcinoma showing patchy opacities in both lungs and sparing of the subpleural space

Whereas colitis is more common with ipililumab therapy, pneumonitis is more commonly seen with PD-1 inhibitors nivolumab and pembrolizumab [23]. According to a meta-analysis including 3232 patients treated with PD-1 inhibitors and 1806 patients with PD-L1 inhibitors (atezolizumab, durvalumab, and avelumab), it seems that PD-1 inhibitors have a higher incidence of pneumonitis compared with PD-L1 inhibitors [27]. According to Khunger et al., the incidence of pneumonitis treated with PD-1 inhibitors was 3.6% (95% CI 2.4–4.8%) as compared to 1.3% (0.8–1.9%) in patients treated with PD-L1 inhibitors. Pneumonitis can be a life-threating complication and therefore needs particular attention. According to the classification of the American Thoracic Society/European Respiratory Society for interstitial pneumonias, a recently published study of 20 patients with nivolumab-induced pneumonitis identified the following four CT patterns[28]: cryptogenic organizing pneumonia (COP) in 65% of patients (Fig. 2), non-specific interstitial pneumonia in 15% of patients, hypersensitivity pneumonia in 10%, and acute interstitial pneumonia (AIP)/acute respiratory distress syndrome (ARDS) in 10%. Radiologists should seek to differentiate pulmonary irAEs from other pulmonary pathologies such as bacterial pneumonias or radiation-induced toxicities. Individually this can be difficult, as the reaction patterns of the lung are limited and show a higher overlap between irAEs and non-irAE pathologies.

**Fig. 3:**
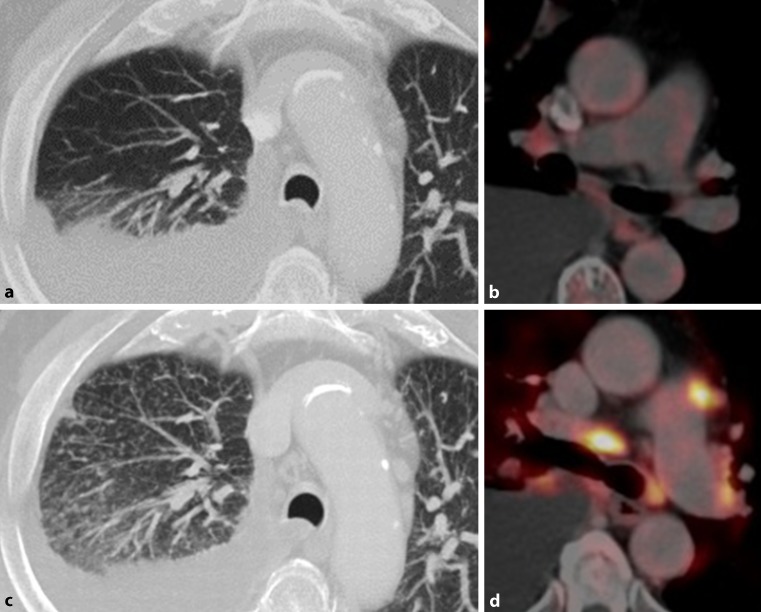
Sarcoid-like reaction in a 77-year-old man with non-small cell lung cancer receiving immune checkpoint inhibitor therapy. **a**, **b** Axial fluorodeoxyglucose positron emission tomography/computed tomography (^18^F-FDG-PET/CT) image obtained before therapy demonstrate a malignant right pleural effusion. **c**, **d** 4 weeks after treatment initiation, numerous intrapulmonary micronodules were detectable in both lungs, predominately right-sided. In addition, enlarged hilar/mediastinal lymph nodes with increased ^18^F-FDG newly developed, consistent with a sarcoid-like reaction. In contrast, malignant tumor burden resolved completely

A special type of irAE is a sarcoid-like reaction that manifests with mediastinal und hilar lymphadenopathy and/or multiple micronodules or ground glass opacities [29]. Imaging features are characteristic; however, in clinical practice, the differentiation from PD, especially in pre-existing malignant mediastinal/hilar lymphadenopathy, might be challenging. In ^18^F-FDG-PET/CT imaging both micronodules and lymphadenopathy can show a considerably increase in FDG uptake (Fig. 3).

## Conclusion

Immunotherapies represent a new class of drugs that have considerably changed treatment strategies in advanced cancers. Based on the special demands of imaging, radiologists become more involved in the care of these patients through the interpretation of staging and restaging examinations. Radiologists have to be aware of the atypical tumor response pattern and common adverse events seen upon imaging of patients under immunotherapy treatment.
